# The weaker sex? Exploring lay understandings of gender differences in life expectancy: A qualitative study^☆^

**DOI:** 10.1016/j.socscimed.2008.05.009

**Published:** 2008-09

**Authors:** Carol Emslie, Kate Hunt

**Affiliations:** MRC Social and Public Health Sciences Unit, 4 Lilybank Gardens, Glasgow, G12 8RZ, UK

**Keywords:** UK, Gender, Lay knowledge, Men's health, Women's health

## Abstract

Despite increasing interest in gender and health, ‘lay’ perceptions of gender differences in mortality have been neglected. Drawing on semi-structured interview data from 45 men and women in two age cohorts (born in the early 1950s and 1970s) in the UK, we investigated lay explanations for women's longer life expectancy. Our data suggest that respondents were aware of women's increased longevity, but found this difficult to explain. While many accounts were multifactorial, socio-cultural explanations were more common, more detailed and less tentative than biological explanations. Different socio-cultural explanations (i.e. gendered social roles, ‘macho’ constraints on men and gender differences in health-related behaviours) were linked by the perception that life expectancy would converge as men and women's lives became more similar. Health behaviours such as going to the doctor or drinking alcohol were often located within wider structural contexts. Female respondents were more likely to focus on women's reproductive and caring roles, while male respondents were more likely to focus on how men were disadvantaged by their ‘provider’ role. We locate these narratives within academic debates about conceptualising gender: e.g. ‘gender as structure’ versus ‘gender as performance’, ‘gender as difference’ versus ‘gender as diversity’.

## Introduction

Studies of ‘lay’ perceptions of health and illness can advance understandings of individual health choices and inform health education and social policy (Blaxter, 1997). Most commentators agree that ‘lay’ people have sophisticated understandings of health and illness, based on intimate knowledge of family members over the lifecourse, social networks and media accounts (Davison, Frankel, & Davey Smith, 1992; Hunt & Emslie, 2001; also see Bury, 1997; Prior, 2003 for useful overviews of the changing status of ‘lay’ perceptions in medical sociology).

Gender plays a key role in lay perceptions of health and health practices. Following West and Zimmerman (1987), we conceptualise gender as a dynamic set of socially constructed relationships embedded in everyday interaction, rather than as a simple attribute of individuals. ‘Doing’ gender means consciously or unconsciously *creating* differences which are then often viewed as ‘natural’ distinctions between men and women. This emphasis on ‘difference’ between men and women creates binary ways of thinking and being. As Annandale and Clark (1996) suggest:

“we artificially, and inappropriately, divide people into two camps…we build a series of other characteristics on top of gender i.e. women are unhealthy, men are healthy; women are irrational, men are rational and so on…real life experience is not like this; attributes and experiences like acting rationally or being healthy cross-cut gender and are not the province of men or women as a group” (p. 22).

Given that “the doing of health is a form of doing gender” (Saltonstall, 1993, p. 12), one way in which men can demonstrate culturally valued (or hegemonic) forms of ‘masculinity’ is by denying vulnerability, taking risks which may injure their health and rejecting health beliefs and behaviours which they associate with women (Connell, 1995; Courtenay, 2000). Whilst there is plenty of evidence to show that many men adopt such strategies, qualitative research supports Annandale and Clark's vision of ‘real life experience’ as being more complex; not all women are eager to consult and not all men are disinterested in their health. For example, a number of studies suggest that women place the health of their families above their own needs, and that a central feature of being a mother is to ‘keep going’ which may involve hiding symptoms and ignoring one's own health (Blaxter, 1983; Pill & Stott, 1982; Popay & Groves, 2000; Walters & Charles, 1997). Studies which explore health in the context of everyday life have often focused on women, but recent work suggests that some men, under certain conditions, resist hegemonic constructions of gender in the way that they talk about health or engage with health care (Emslie, Ridge, Ziebland, & Hunt, 2006; O'Brien, Hunt, & Hart, 2005; Robertson, 2007). In addition, the resources people have for constructing gender vary by socio-economic status, sexuality, ethnicity and other markers of social position.

These strong links between the acting out, and (re)creation, of gender differences and health suggest that there is much to be learnt from examining lay understandings of gender differences in health. In this paper, we are interested in exploring lay understandings of gender differences in mortality as we are not aware of any qualitative studies which focus on this topic. This neglect is interesting, given that in virtually every society in the world, women now have a longer life expectancy than men (Barford, Dorling, Smith, & Shaw, 2006). A few quantitative studies of perceptions of gender and life expectancy have been conducted, but the results are contradictory. In two studies, participants correctly perceived that women in the UK had longer life expectancy than men. Macintyre, McKay, and Ellaway (2005) found that 88% of women and 87% of men in a general population sample in Scotland indicated that women lived longer than men, while Popham and Mitchell (2007) found that a significantly higher proportion of men than women in the British Household Panel Survey believed that they were ‘not likely’ to live to 75 years. By contrast, a study of students in the United States (Wallace, 1996) did not find gender differences in young men's and women's estimates of their personal life expectancy. However, in response to an open-ended question about reasons for women's greater longevity, a higher proportion of female than male respondents attributed this to women taking better care of their health, while more male than female respondents attributed this to the physical demands of men's jobs. Only a small proportion of men and women (16% and 14% respectively) attributed the gender gap in life expectancy to biological factors. In-depth qualitative research can illuminate the reasoning and complex meanings attached to such perceptions. Below, we briefly review current hypotheses on gender and mortality before outlining our qualitative study.

Gender differences in mortality are influenced by both socio-cultural and biological factors, although the extent to which each makes a contribution varies for different health conditions (Krieger, 2003; Wizeman & Pardue, 2001). Bio-medical research has investigated biological differences between men and women in anatomy and physiology (particularly related to the reproductive system) and in a wide range of metabolic and hormonal factors and, whilst these biological differences are clearly important in shaping patterns of morbidity and mortality, they are usually considered quite separately from the social environment. Conversely, sociological research on patterns of illness “treats biology as socially neutral and builds on the assumption that inherent biological differences between men and women are either minimal or largely irrelevant” (Bird & Rieker, 1999, p. 107). In other words, “the biological” is explicitly played down (Birke, 2000). Furthermore, it has been suggested that interconnections between sex and gender, or the biological and cultural, might be typified as the *gendered expression of biology* when biological difference, such as reproductive capacity, influence gender divisions (making women responsible for looking after children because they have given birth, for example) or as the *biologic expression of gender* when gender divisions themselves are expressed in the biological body. In sum, despite an obvious need to understand when and how, or indeed whether, biology (sex) matters for a particular health outcome, very little research on gender and health has attempted to integrate biological and sociological models of pathogenesis or salutogenesis.

Variations in gender differences in life expectancy simply illustrate the complexities of the link between the biological and the social. The World Health Organisation notes that women's “innate constitution appears to give women an advantage over men, at least in relation to life expectancy. When this female potential for greater longevity is not realised it is an indication of serious health hazards in their immediate environment” (World Health Organisation, 1998). Before birth, sex manifests itself in higher male foetal loss and vulnerability to external maternal stresses (Kraemer, 2000). The complex ways in which this apparent greater biological vulnerability of males is then mediated by gender (the different social realities of being male or female in different contexts) is illustrated by the huge variation in sex differences in average life expectancy (LE). World-wide LE is 65 years for men and 69 for women (World Health Organisation, 1998), but sex differences in LE are smallest in countries where LE is lowest and currently highest in countries of the former Eastern block. These countries illustrate how social and political changes can have a profound impact on sex differences in health even within a short time frame: for example, between 1987 and 1995 the sex difference in LE in Russia increased from 9 to 14 years (Chenet, 2000).

Socio-cultural explanations for women's increased longevity generally draw on traditional gender roles and social constructions of ‘masculinity’. There is some debate over whether traditional social roles benefit men or benefit women. While the traditional male ‘provider’ role has put men at greater risk than women of premature death from accidents and exposure to occupational hazards (Doyal, 2000; Waldron, 1983), traditional gender roles advantage men as they benefit from socially constructed inequalities including better access to health-related resources (Doyal, 2000). In addition, men still occupy higher status, higher paid jobs and benefit from the gendered division of labour, and women may suffer more role conflict than men, given that many work a ‘double shift’ in the public and the domestic sphere (Bird & Rieker, 1999). There is some debate about whether gender differences in mortality will reduce as men's and women's lives become more similar (e.g. increasing gender equality and a more similar distribution of social roles and health-related behaviours) (Backhans, Lundberg, & Mansdotter, 2007).

In this paper, we explore lay explanations for women's longer life expectancy and investigate whether there are gender differences in these perceptions. Because changes in gender roles and in health-related behaviours may influence experiences and perceptions, we have chosen to compare the accounts of respondents in two generations (aged 30 years and 50 years).

## Methods

Respondents were sub-sampled from the youngest (born in the early 1970s) and middle (born in the early 1950s) cohorts of the West of Scotland Twenty-07 Study, a longitudinal survey of the social patterning of health (Ford, Ecob, Hunt, Macintyre, & West, 1994). Semi-structured interviews were conducted with 11 men and 11 women in their early fifties, and 11 men and 12 women in their early thirties (described here as ‘50s’ and ‘30s’, respectively) as part of a broader study of age, health and constructions of gender. We used purposive sampling (Mays & Pope, 1995) in order to achieve a diverse sample in terms of social roles, self-rated health and gender role orientation. Half of the sample were selected to be typical of their age in certain respects; they were married or cohabiting, were parents *and* perceived themselves to be in reasonable health. The other half had less ‘conventional’ biographies (e.g. never married, never had children, perceived their health to be poor, or had extremely high or low scores according to a measure of gender role orientation) (Bem, 1981; see Emslie & Hunt, in press, for further details). Although our main focus was on gender, we sought to sample men and women from a range of socio-economic positions. All, with one exception, were from the ethnic majority white population, reflecting the relatively homogeneous ethnic composition in this area. Ethical permission for the qualitative study was granted by the University of Glasgow Ethics Committee for non-clinical research involving human subjects.

After an explanation of the study and assurance of confidentiality, respondents were asked to give informed consent and permission for their interview to be tape recorded. In order to attempt to access the relationships and assumptions that made up the respondents' worldview (McCracken, 1988), respondents were asked first to give a brief overview of their life. Using this overview as a guide, the interviewer then concentrated on particular stages of their biography, on decisions around work-life balance and on health. This paper focuses on discussions around gender and health. Respondents were informed that life expectancy had increased over time for both men and women, but that women's life expectancy was still around 5 years longer than men's, and asked if they had any explanations for this difference. Respondents sometimes elaborated on these explanations elsewhere in the interview – for example, when they were asked whether they believed men and women had similar or different attitudes to their health – and when this occurred, these responses were also included in this analysis.

Interviews were transcribed, and transcripts were checked against the tapes. Preliminary analysis began during fieldwork, with interviews being conducted in batches and discussed before further interviews were set up. Some questions were modified in the light of these discussions. The software package QSR Nvivo was used to facilitate analysis. Following McCracken (1988), analysis moved from the particular (a detailed analysis of language in each transcript) to the general (a comparison of patterns and themes across all the transcripts). Hypotheses were formulatesd, tested against the transcripts, and where necessary re-formulated in a cyclical process. In the interview extracts below, all names are pseudonyms and ‘50s’ refers to those respondents in the 1950s cohort (in their early fifties) while ‘30s’ refers to those in the 1970s cohort (in their early thirties).

## Findings

None of the respondents seemed surprised by the statement that women had a longer life expectancy than men; many agreed (‘aye, men don't have as long’) or even interrupted the interviewer to complete the end of the sentence. However, respondents generally found it difficult to explain *why* women lived longer than men; many were puzzled because they believed that the risks of childbirth should disadvantage women, or because the factors which had disadvantaged men in the past (e.g. hard manual work or heavy drinking) had changed in recent years. Male respondents jokily enquired how they could avoid this fate (‘I'd like to know so I can try to avoid it myself!’) or drew on traditional gender stereotypes (‘Maybe it's just to annoy men!’) before admitting that they found women's longevity difficult to explain. The few female respondents who used humour in their explanations for differences suggested it was because women worked harder than men, or deserved a break after men ‘pop off’. Only one respondent commented, albeit jokingly, on the ‘injustice’ of women's greater longevity and questioned the appropriateness of the term ‘the weaker sex’:

WILL^1^ (50s): I don't know why, but I know it really annoys me the fact that they do, and the fact that their pensionable age is five years in the opposite direction!…I've often worried about the injustice of that…whose idea it was…the weaker sex!

Respondents' accounts of women's longer life expectancy were multifactorial. Biological explanations were less common, much less detailed and more tentative than socio-cultural explanations. We describe each in turn.

## Biological explanations

Only half of the sample drew even tangentially on biological explanations to explain gender differences in life expectancy; there were no discernable differences by generation. These accounts ranged from general descriptions of women being ‘tougher’, ‘stronger’, having a different ‘makeup’ or having increased ‘stamina’ compared to men, to narratives which drew on scientific terms such as ‘hormones’, ‘testosterone’, ‘oestrogen’, ‘chromosomes’ and ‘genetics’. References to biology were usually tentative and brief (e.g. ‘maybe it's a genetic thing’, ‘maybe women are just tougher’) and almost always combined with socio-cultural explanations. Only two respondents presented a solely biological explanation without considering any socio-cultural factors; both were in the younger cohort, and one had a chromosomal disorder and so had personal knowledge of the importance of ‘genetic makeup’.

A small group of men (all University-educated professionals) were unusual in considering biological explanations in more detail. For example, Alec discussed interactions between biology and environment. He argued that women's ‘hormones’ – like men's – ‘drove’ them towards dangerous behaviour, but that women had only recently yielded to these ‘drives’ because of changing cultural attitudes. Johnny also considered both environmental and biological factors before concluding that women's biology (‘body fats’) protected them from the effects of stress:

ALEC (50s): (Men have) all the testosterone that drives you to do these (dangerous) things (which) can be tempered by environmental influences…Women…they've got hormones as well (laughs), that drive them to do things…You can see younger women now…going on pub crawls…and getting into scrapes whereas in my generation you didn't see that sort of thing. So there's that environmental influence influencing THEM now…These women will be having heart attacks and burnt out livers the way men had…20, 30 years ago.

JOHNNY (30s): Presumably eventually the same number of women (as men) will drop dead of stress – unless they're better equipped – and I've seen stuff that says women are better at coping with stress because it's something to do with body fats. I don't think it's as simple as women had it easier, they lived longer, cos it's just not true…I've got a sneaky suspicion it might be a biological imperative, and that's a reason why women live longer than men.

Female respondents also alluded to interactions between social and biological factors when they attributed women's increased longevity to their potential role as mothers. For example, Michelle drew on her experience as an auxiliary nurse to note women's quicker recovery from heart operations which she ascribed to their need to tolerate pain during childbirth, while Sharon suggested that women' s ‘overactive’ hormones influenced their ability to stay calm when dealing with the constant demands of home, children and paid work. Both women moved seamlessly between references to biology (‘in their makeup’, ‘hormones’) and gendered social roles (‘men have had everything done for them’, ‘women…dealing with the kids, with work, with the house’):

MICHELLE (50s): People that come for the heart operations…the women get on better. I think it's just in their makeup, they sort of shrug it off and ‘right I am going to get on with this’ whereas the men…in that generation they will have had everything done for them…Women have the children and they are just used to getting up and get on with it whereas men, if men had the children…! The pain threshold in men is worse than women…much lower.

SHARON (30s): (Women live longer because of) overactive hormones (laughs)!…He (partner)…gets too worked up…whereas I am…dead laid back and just get on with it. I think probably most women are like that…dealing with the kids, with work, with the house…whereas…older men they were used to just going out and going to their work and coming in and getting their dinner…I think it keeps us going…if my ma (mother) stopped, she would collapse.

## Socio-cultural explanations

All but two people drew on socio-cultural factors to explain women's greater longevity and these accounts were often detailed and assured. Many respondents set these explanations within a wider structural framework, talking about general increases in life expectancy due to advances in medical science, improvements in public health, reductions in family size and increases in health education over time. Respondents drew on three socio-cultural explanations for men's lower life expectancy: traditional gender roles, cultural restrictions on men admitting to symptoms and consulting health professionals, and men's poorer health-related behaviours. These three explanations were linked by the perception that social change would influence patterns of longevity; thus as men's and women's lives converged, so too would life expectancy.

### Traditional gender roles

#### Manual work and the strain of the breadwinner role

Men's greater participation in paid work (especially heavy manual work) was perceived to have contributed to their shorter life expectancy in previous generations. While women tended to refer briefly to this explanation, it was discussed more often, and in more detail, by men (particularly older men). Men suggested that women benefited by lesser participation in the labour market (conceptualised as encompassing stressful, dangerous and competitive environments), although they were careful to acknowledge the challenges of caring roles (‘it's a hard job looking after a big family’).

Some respondents suggested that the strain of the breadwinner role also disadvantaged men. Will considered different occupational experiences before suggesting that the anxiety caused by being out of work – which he believed to be more serious for men – could be part of the explanation. Similarly, Annie suggested that men still felt they had the main responsibility to provide for their families, even when both partners were working, and that this stress could explain their shorter life expectancy. Her use of the terms ‘inbred’ and ‘inbuilt’ to describe these deep-rooted feelings of responsibility could be interpreted as either a biological or social (through upbringing) explanation:

WILL (50s): Traditionally…PERHAPS they (women) had an easier life in so far as they didn't go down coal mines (or) on shipyards…but it can't just only be that. And I wonder if it has to do with anxiety…A man's got to work, if a man's not at work…that can be a SERIOUS anxiety problem. It's an anxiety problem for the woman, but I don't think she's just as anxious as the man.

ANNIE (30s): Men are just generally more stressed out than women…they kind of take it on their shoulders that if anything goes wrong then it's them to blame and not you as a couple. Maybe it's…inbred in them…because, going back many, many years ago it was…the man that always went out to work…maybe it's just a kinda inbuilt thing with them that they feel responsible.

#### Caregiving roles

Half of the female respondents referred to women's roles as homemakers in their explanations of gender differences in longevity. They felt that while men could relax when they came home from work, knowing their wife had their dinner ready for them, women were constantly occupied. Women's longer life expectancy was thus explained in terms of the need for caregivers to be innately tougher as they juggled multiple roles (see extracts from Sharon and Michelle quoted earlier) or, as Fiona suggests, through the benefits they received from this constant physical activity:

FIONA (30s): Certainly that (older) generation, they did have it hard. OK, maybe the men went down the mines…But at the end of the day, they just came in and…didn't have to do much else and they were waited on hand and foot. Whereas I don't think the women ever really stopped…Maybe they have more physical exercise.

However, there was some confusion about whether women's health would be *damaged* through this constant work. For example, May was unsure whether women's constant activity after retirement would benefit or disadvantage their health:

MAY (50s): I would have said if anything the WOMEN would have gone first…They (men) seem to all just relax and the women (are) doing all the (domestic work)…Maybe it's that that keeps them going!

### Men's reticence to discuss health problems and seek help

Just under half of the sample referred to men's inability in caring for their health in their explanations of gender differences in longevity; this explanation was more common among women than men. Both men and women made frequent references to the stereotypical notion that men will not go to the doctor or talk about their health. Some narratives had a critical tone:

RONA (50s): Men just are so apathetic…they have to be sort of dragged (to the doctor's)…I don't think they want to admit that there could be anything wrong with them.

Respondents also suggested that women coped better with stress by talking to friends or expressing emotions, while men hid problems and tried to pretend everything was fine because of constraints on male behaviour (‘a man thing’, ‘the macho thing’). This ‘bottling up’ of stress was believed to have adverse consequences for men's health:

JIMMY (50s): I think they (women) cope with things more easily…they would talk about it. I feel as if men, I know they do hide a lot of things…I suppose it's just pride…or it's a man thing.

LESLEY (30s): Men…it's still seen as the macho thing that they cannae talk to their best friend if something's bothering them, they've not really got anybody to go and speak to…I know like stress…if you let it bottle up…it has a detrimental effect on your health.

A number of men developed this theme, talking in some depth about the adverse consequences of ‘macho’ or ‘masculine’ behaviour for men's health. One man confined this ‘dangerous’ masculine behaviour to one particular part of the lifecourse (youth) and suggested that once men got to their mid 30s ‘the danger decreases’:

ALEC (50s): Being a young man is, I think, is very dangerous to your health…Playing sports…going out drinking…threats of violence…I think the danger decreases and I think it kind of levels out…it's equally balanced between men and women…when you get to an age of about 35…The danger…comes in men's health…if they carry on doing the same things as they did when they were 20.

Similarly, two younger men discussed how ‘macho’ behaviour led to men ignoring illness. Both set their explanations in a Scottish context and discussed the difficulties that men had talking about personal matters:

GAVIN (30s): We're terrible in Scotland for talking about personal things…I think a lot of it just comes from the psyche of men…(At work), the women are having conversations, have a very active social group…and then there's a group of sad men in the corner with nothing to say to each other.

AL (30s): Maybe I'm generalising but I don't know if it's a sort of Scottish thing…you have to actually be DYING before we (men) contemplate taking time off work or go to see a doctor…On the other hand when we do get ill – even if it's just a heavy cold – we're the bane of the women in our lives' existence…Men have a greater difficulty talking about health problems, and do have more of a sort of the head in the sand approach, that if you ignore it, it'll go away. It's a very sort of masculine attitude. Although it's said that men – you know, the sort of new man is sort of more in touch with himself and…you don't want to be a hypochondriac either…I don't know if it comes back to the sort of old sort of rules of being macho…that…illness is a weakness…Whereas with women, it's not any big deal.

Al's account also points to some contradictions within ‘common sense’ understandings of men's attitudes to health: for example, men won't go to the doctor until they are ‘dying’ and are worried about being seen as ‘hypochondriacs’ but will moan if they have a cold. It is also interesting that he referred to the ‘rules of being macho’, suggesting he believed these to be constructed conventions, rather than ‘natural’ behaviours. Al's conceptualisation of ‘rules’ also allowed some lee-way for variation; indeed he noted men may approach health in different ways (‘masculine’ versus ‘new man’), although he distanced himself somewhat from this assertion (‘it's *said*…that the new man is more in touch with himself’).

Similarly, other respondents presented a more complex picture of men and help-seeking and many acknowledged that they were generalising about ‘men’ and ‘women’ and that there were always exceptions to the rule. For example, Sophie acknowledged that she was generalising about women being quicker to seek help than men, and went on to reveal that she had delayed going for a cervical smear test:

SOPHIE (30s): It's a generalisation here but I think if a women was to find a lump in her breast, she would do something about it quickly. I think a guy would wait longer…But then again…thinking about myself here, I'm criticising blokes and it's something I do myself. I've had about three letters to go for a smear test and I still never went.

Other respondents, particularly in the younger cohort, gave specific examples of men who *did* care for their health. Male respondents, particularly those from professional backgrounds, tended to contrast traditional ‘macho’ men, who don't look after their health, with other groups of men who do (see Al's reference to ‘new men’ above). For example, Eddie contrasted the attitude of ‘macho’ men who believe that healthy eating is for ‘wusses’, with guys who go to the gym and students who look after their bodies, while Keith compared men who had a problem talking about health (‘hard men’, ‘Highlanders’^2^) with men he met at Art College, who perhaps had different ways of ‘doing’ masculinity and were less concerned to be perceived as strong:

EDDIE (30s) Amongst certain groups of blokes, healthy eating and all that is seen as for wusses…on the other hand, lots of guys go to gyms now and look after themselves…In the student population, there's more implicit emphasis on looking good and being fashionable and looking after your body.

KEITH (30s): Health's just another thing that you're having to open up about so …if guys have got a problem talking about health they've got a problem talking about everything…A lot of guys are concerned about how they're being perceived, if they're being perceived to be strong…(Like the) Highlander…which can lead to disaster, like a drink problem…I think the people that I met at Art College are generally different …to some of the guys that I'd have gone to school with…(who) are still trying to act the hard man.

Women also acknowledged that men were not a homogenous group, but tended to frame this in terms of change over time. They suggested that young men were more interested in health than older men because of the increased availability of health information and decreasing pressures on men to be ‘macho’. For example, Fiona and Heather suggested that attitudes to health were changing over time for men, although they had reservations about how much they would change in the ‘macho’ west of Scotland:

FIONA (30s): Men are more aware of their health now…there's posters up all over the place and there's more Wellmen's clinics…More of them are going to the gym and looking after their health and eating healthily, not in the west of Scotland right enough…There's a gap between…younger men and older men.

HEATHER (50s): I think it's changing for men…I was looking for a magazine for my son…, saw “Men's Health”…and thought he would probably pooh pooh me …and he said “that's a really good, good magazine mum”…so I think maybe the younger generation of men hopefully will be more at ease than the older. Unfortunately there is still that macho West Coast of Scotland macho image.

### Smoking and drinking

Half of the men in the sample, compared to many fewer women, suggested that smoking or drinking might account for gender differences in longevity. Only one older woman referred to the possibility that ‘lads' culture’ (“smoking and drinking, watching football and stuffing your face”) could adversely influence men's life expectancy but she quickly dismissed this explanation (“I'm talking out of the top of my head there. I don't actually know any men who sit and smoke and drink and watch TV, but I believe it exists!”). Four younger women suggested that, in the past, men drank or smoked more than women; they all believed that this was changing and linked this convergence in health behaviours to changes in gender roles (e.g. women having increased access to higher education and better careers, more disposable income and spending more time in the public sphere). For example, Sophie linked changes in women's smoking and drinking patterns to more general changes in gender relations:

SOPHIE (30s): Maybe alcohol is a factor but I think women are now beginning to catch up on men in that respect…There's more women now smoking than men…it's getting through to blokes now and you'll actually see a shift in patterns…In the past, women would have never went to bars unless…they were out on a date with a guy. But now…we would think nothing of going out with our pals. Women are at University now, I mean there's so many changes in…social circumstances and I think a lot of that's to do with women out working as well, rather than being in the home and being just a mother.

Amongst men, excessive male drinking was linked to the need to display ‘macho’ behaviour (see Keith's earlier comment about ‘Highlanders’). One man suggested that machismo and peer pressure led to the idea that “you're not a man if you don't have another pint” (Eddie, 30s). However, many men also suggested that gender patterns in smoking and drinking were changing. For example, Johnny set changes in men's drinking culture within a structural context, describing how the ‘heart attack generation – predominantly male middle managers who lived through the stresses of organizational change in the 1980s – would be ‘dropping dead in their fifties’:

JOHNNY (30s): There's a whole generation of them…dropping dead in their fifties because of the stress they put themselves through in the ‘80s…I suspect…it is more men because I think the drinking culture…men were living harder at that point…When I went out from work these days, it is likely to be in mixed company or company where there are more women than men and pretty much drinking the same amount…but that's a new phenomenon.

Another man drew on examples from his working life as an engineer and suggested that the material conditions of life, as well as diet and alcohol use, among men who worked as labourers in the west of Scotland contributed to their early death from heart disease:

GARY (50s): The west of Scotland, what men do, their diet and their lifestyle doesn't help (their survival)…You came across guys…labourers…and they'd have a can of beer before they got started (work)…I know a lot of these people either they didn't get to retirement age or…within a few years of being retired they'd passed away…It's maybe too big a brush to say, this is so prevalent in the west of Scotland…but I think a lot of it was down to people's diets…at one time we were the highest cardiac…rate in the country…Now what is it causes that…social stresses, housing, living standards, what? All these things all add together but I tend to think that…a lot of it's what people diverted on their earnings, what they did with it.

## Discussion

This qualitative study sought to address a gap in the literature on lay perceptions of gender differences in mortality. There were no substantive generational differences in findings and, with few exceptions, male and female respondents gave similar explanations for women's longevity. Our data suggest that respondents were aware of women's increased longevity, but found this difficult to explain. Socio-cultural explanations were more common and detailed than biological explanations, although respondents sometimes gave sophisticated explanations which combined both perspectives (Bendelow, 1993). Other studies (Emslie, Hunt, & Watt, 2003; Richards, 1996) have found that ‘lay’ discussions of biological processes are often tentative, brief and characterised by uncertainty, perhaps reflecting a general lack of understanding and confidence about discussing science. In contrast, socio-cultural explanations more easily incorporate ‘lived experience’ and so respondents may have felt more ‘expert’ and able to draw on their own observations and/or experiences of changes in gender relations and health practices such as smoking and drinking. It is interesting that respondents' relative neglect of biological explanations for women's longevity mirrors the ‘playing down’ of the biological by sociologists.

Frankel, Davison, and Davey Smith (1991) have argued that individuals understand and interpret health risks ‘through the routine observation and discussion of cases of illness and death in personal networks and in the public arena’, in a process they call ‘lay epidemiology’ (p428). Like them, we found many parallels in the explanations that ‘lay’ and ‘professional’ epidemiologists advance for gender differences in mortality; both groups propose multifactorial theories drawing on socio-cultural and biological factors, hypothesise about the convergence of traditional gender roles leading to a reduced gender gap in mortality, and emphasise the importance of gender differences in health-related behaviours. Experiences of health and death among relatives are particularly salient for ‘lay’ epidemiologists, not just because of their emotional resonance, but also because the family offers an opportunity for in-depth, lifecourse observation of exposures, behaviours and outcomes (Hunt & Emslie, 2001). The observation of structures and behaviours believed to influence mortality (e.g. the domestic division of labour, ways of ‘doing’ gender and health-related behaviours within close social networks) is a vital component of ‘lay’ understandings of health and death, as well as being important for the construction of gendered identities.

There were also parallels between our data and ‘expert’ theories on social class inequalities in health. First, the way that respondents drew on gendered social roles to explain women's increased life expectancy in terms of differential exposure to hazards has parallels with both ‘hard’ and ‘soft’ structural models (Macintyre, 1997). Our interpretation of respondents' narratives suggested that the physical, material conditions of life (determined both by the gender order and occupational class position) could either directly influence gender differences in health and death (e.g. working-class men are exposed to occupational hazards and accidents at work) or indirectly influence gender differences (e.g. men's breadwinning role leads to increased ‘stresses and strains’ which result in heart attacks). Secondly, the ways that respondents drew on men's poor health behaviours to explain differences in life expectancy has parallels with behavioural explanations of class inequalities (Macintyre, 1997). ‘Hard’ behavioural models centre on the behaviour of men (e.g. men ‘choose’ to be ‘apathetic’ about their health and/or take part in ‘risky’ behaviours), while ‘soft’ behavioural models view these individual behaviours as being embedded within social structures (e.g. men's individual behaviours are influenced by cultural constructions of masculinity which emphasise risk-taking or are located within the context of poor health behaviours in the west of Scotland). Finally, there were intersections between structural and behavioural models; for example, changes in gendered social roles were linked to changes in smoking and drinking which in turn influence gender differences in mortality.

Our findings have also resonance with key debates in the academic literature on gender. Our data reflect academic debates about ‘gender as difference’ as opposed to ‘gender as diversity’ (Annandale & Hunt, 2000). With regard to the former position, respondents in our study subscribed to a collective narrative that women were inherently ‘tougher’ than men and that men were more vulnerable to stress (Bendelow, 1993; Pietila & Rytkonen, 2008). These cultural discourses are interesting as they seem to invert popular understandings of women as the ‘weaker’ sex. However, Bendelow (1993) suggests that this perception may be “double-edged” for women: “the assumption that they may be able to ‘cope’ better may lead to the expectation that they can put up with more pain (and) that their pain does not need to be taken so seriously” (p. 287). To some extent, it could be argued that our line of questioning – asking for explanations for gender differences in mortality – may have lead respondents towards explanations which emphasised *difference* between men and women. However, narratives sometimes incorporated subtle discussions of diversity, which resonate more closely with academic discussions of “a multiplicity of masculinities and femininities inhabited and enacted variously by different people and by the same people at different times” (Paechter, 2003, p. 69).

These ‘lay’ explanations also reflect the tension between ‘gender as structure’ versus ‘gender as performance’ (Hunt, 2007). The importance of ‘gender as structure’ was evident from the emphasis given to the gendered division of labour (male ‘breadwinner’ versus female ‘caregiver’), even though respondents acknowledged that this was more a feature of previous generations than of contemporary life and were keenly aware of recent changes in gender relations. Female respondents were more likely to focus on women's reproductive and caring roles, while male respondents were more likely to focus on how men were disadvantaged by their ‘provider’ role (Wallace, 1996). Given that narratives can be viewed as a way of asserting ‘moral worth’ (Blaxter, 1997), it may be that each gender prioritised their (traditional) contribution to society. It is particularly interesting that female respondents focused on women's hard domestic lives, given that they were asked to explain women's mortality *advantage*; perhaps their narratives about the domestic division of labour functioned as a way to reassert the lived (unequal) experiences of women. Respondents' narratives also reflected the notion of ‘gender as performance’. For example, discussions around alcohol could clearly be interpreted as ways of ‘doing’ gender (West & Zimmerman, 1987). However, it is important to note that gender ‘performances’ were usually located within wider structural contexts such as cultural norms associated with masculinity, changes in gendered social roles or wider geographic and socio-economic contexts (Williams, 2003). As Popay and Groves (2000) suggest, “Narrative accounts of experiences …illuminate the subjectively experienced relationship between identity…agency…and social structures…which impinge on the ways in which individuals negotiate/live their lives” (p. 76). Future work on ‘lay’ perceptions of gender and health is necessary in order to illuminate the complex interplay between rapidly changing gender identities, embodied experiences and structural inequalities between men and women.

## Footnotes

1 For those born before 1950 in the UK, the State Pension age was 60 years for women and 65 years for men. The State Pension age for women will increase gradually from 2010, so that by 2020 it will be 65 for both men and women.

2 Stereotypes of people from the Scottish Highlands often emphasise heavy drinking and emotional stoicism.
